# A Bayesian nonparametric method for prediction in EST analysis

**DOI:** 10.1186/1471-2105-8-339

**Published:** 2007-09-14

**Authors:** Antonio Lijoi, Ramsés H Mena, Igor Prünster

**Affiliations:** 1 Department of Economics and Quantitative Methods, University of Pavia, 27100 Pavia and Institute for Applied Mathematics and Information Technology, National Research Council, 20133 Milan, Italy; 2 Research Institute for Applied Mathematics and Systems, National Autonomous University of Mexico, Mexico City, A.P. 20-726, Mexico; 3 Department of Statistics and Applied Mathematics and ICER, University of Turin, 10122 Turin and Carlo Alberto College, 10024 Moncalieri, Italy

## Abstract

**Background:**

Expressed sequence tags (ESTs) analyses are a fundamental tool for gene identification in organisms. Given a preliminary EST sample from a certain library, several statistical prediction problems arise. In particular, it is of interest to estimate how many new genes can be detected in a future EST sample of given size and also to determine the gene discovery rate: these estimates represent the basis for deciding whether to proceed sequencing the library and, in case of a positive decision, a guideline for selecting the size of the new sample. Such information is also useful for establishing sequencing efficiency in experimental design and for measuring the degree of redundancy of an EST library.

**Results:**

In this work we propose a Bayesian nonparametric approach for tackling statistical problems related to EST surveys. In particular, we provide estimates for: a) the coverage, defined as the proportion of unique genes in the library represented in the given sample of reads; b) the number of new unique genes to be observed in a future sample; c) the discovery rate of new genes as a function of the future sample size. The Bayesian nonparametric model we adopt conveys, in a statistically rigorous way, the available information into prediction. Our proposal has appealing properties over frequentist nonparametric methods, which become unstable when prediction is required for large future samples. EST libraries, previously studied with frequentist methods, are analyzed in detail.

**Conclusion:**

The Bayesian nonparametric approach we undertake yields valuable tools for gene capture and prediction in EST libraries. The estimators we obtain do not feature the kind of drawbacks associated with frequentist estimators and are reliable for any size of the additional sample.

## Background

Expressed Sequence Tags (ESTs) are generated by partially sequencing randomly isolated gene transcripts that have been converted into cDNA. From their introduction in Adams *et al*. [1], ESTs have played an important role in the identification, discovery and characterization of organisms as they provide an attractive and efficient alternative to full genome sequencing. The resulting transcript sequences and their corresponding abundances are the main focus of interest providing the identification and level of expression of genes. Important issues to be addressed in terms of design of a future study are: (1) a comparison among different cDNA libraries of the same organism with the aim of detecting the least redundant library; (2) the determination of an "optimal" number of genes to be sequenced by the experimenter. Indeed, these issues are relevant since, despite the novel advances in technology, see [2], sequencing is still expensive and therefore suitable cost-effectiveness thresholds must be established. This suggests that there is the need for assessing the relative redundancy of various libraries prepared from the same organism in order to detect which one yields new genes at a higher rate. Indeed, there are 'normalization' protocols which aim at making the frequencies of genes in the library more uniform thus typically improving the discovery rate. However, performing such protocols is also expensive. Hence, the decision, whether to proceed with sequencing of a non-normalized library or to resort to a normalization procedure, has to balance carefully the involved costs: such a decision is necessarily based on statistical estimates of the coverage of the given sample, of the expected number of new genes in a future sample and on the future discovery rate. Note that ideally one would like to sequence the smallest possible portion of the library and, based on the outcome, predict the tentative future sequencing well beyond the size of the given dataset.

The practical issues discussed above naturally translate in statistical problems which, given an initial sample of EST, can be described as follows:

a) *Coverage*: Coverage can be seen as the proportion of genes in the library represented in the initial sample or, equivalently, the probability that a new read will not produce a new gene. The coverage estimate provides a first description of redundancy of the library.

b) *Expected number of new genes*: Having observed an initial sample of size *n *generated from the cDNA library and estimated its coverage, prediction of outcomes of further reads is in order. The first question to answer is: 'How many new unique genes are expected to be detected in an additional EST dataset of targeted size *m*?' Such estimates provide, then, an overall measure of redundancy of the library with reference to a further EST survey.

c) *Discovery rate*: In addition to the expected number of genes in a future sample of size *m*, it is also important to establish the rate at which the probability of discovering a new gene decays as more and more reads are recorded. In other words, interest lies in determining the probability that the (*n *+ *m *+ 1)-th read leads to a new gene, given the observed initial sample of size *n *and regardless of the experimental outcome yielded by the *m *intermediate draws. The availability of the discovery rate as a function of the size of the future sample *m*, represents then a pointwise predictive measure of the evolution of redundancy as the sequencing ideally proceeds.

Note that the combination of the measures under b) and c) provides a natural guideline for selecting the size of a future sample *m*. Supposing the targeted number of new genes is *j*, the estimator in b) guides the selection of the minimum sample size $\bar{m}$ which should lead to *j *new genes. Then, one can resort to the discovery rate: in case it is relatively low around $\bar{m}$, it may be convenient to reduce the size of the future sample in a way that the discovery rate does not fall below a threshold suggested by the problem at issue. In such a case, one obviously would not be able to achieve the targeted number *j *of newly observed genes: costs considerations may indeed suggest that, with such a low discovery rate, sequencing is too expensive. On the other hand, if the discovery rate around $\bar{m}$ is still relatively high, one may decide to enlarge the survey size. Moreover, the information conveyed by b) and c) is useful in comparing libraries and, again, it is worth considering these estimates together. Indeed, suppose we have to compare two libraries and that, for a fixed size *m *of the additional sample, library 1 yields a larger expected number of new genes but a lower discovery rate in comparison with library 2. If the sample size *m *is increased to *m *+ *m*', for *m*' sufficiently large, the comparison between the two libraries can lead to different conclusions in the sense that a larger number of new genes is predicted for library 2. This happens because library 1 features a lower discovery rate, which implies that, within the additional *m*' draws, the expected number of new genes is lower for library 1. With reference to 'normalization' protocols, this means that the decision whether to carry it out or not should also depend on the foreseen sample size. For instance, the normalized *Mastigamoeba balamuthi *data we analyze exhibit a higher discovery rate, with respect to the non-normalized one, for small *m*. But, since the discovery rate has a faster decay, it appears that, already for moderately large *m*, the effect of the 'normalization' is exhausted producing fewer number of new genes. The three questions raised above can be seen as particular instances of classical species sampling problems: indeed, in the present context each species takes on the meaning of gene and the population is given by the library. Species problems appear in a variety of different applied situations such as astronomy, ecology, linguistics, machine learning, population biology. We now briefly recall well-known estimation methods which have recently been applied to EST data and then outline the key ideas of our Bayesian nonparametric approach.

### Estimation methods

The main frequentist tools, that are useful for inference on the cDNA library properties described in the previous section, are based on the theory set forth in Good [3] and Good and Toulmin [4], where nonparametric estimators for the sample coverage and the expected number of new species to be detected in a future sample of size *m*, given the initial sample, are provided. The estimator of the sample coverage in [3] coincides with the proportion of distinct species represented by at least two units in the sample. Good attributes the original idea to Turing and this explains why it is usually referred to as Turing estimator. The popular Good-Toulmin estimator for the number of new species to be observed in a future sample is derived in [4] and, as a by-product, an evaluation of the discovery probability is achieved. Recently, the interest in species sampling problems has remarkably grown, mainly due to their importance in genomics. Indeed, Mao [5] studies various properties of the Good-Toulmin estimator and shows that it can be also viewed as a nonparametric empirical Bayes estimator. In [6], the authors suggest a parametric variation of the Good-Toulmin estimator. An alternative to it is presented in [7], where the detection of ESTs from each gene in EST sequencing is modeled by means of a Poisson process whose intensity is governed by some unknown distribution. It is to be noted that all frequentist nonparametric approaches lead to reliable estimates for the number of new genes in an additional sample only if its size is not too large. For instance, if the size of the additional survey *m *is larger than the initial sample *n*, it is well-known that the Good-Toulmin predictor can become a monotone decreasing function of *m*: this leads to the paradox of predicting fewer new genes by enlarging the additional sample size *m*. Even the nonparametric alternative proposed in [7] yields reliable results only when *m *≤ 2*n*. This fact is also outlined in [8]. Hence, if one wishes to predict the number of new genes for large *m*, one needs to resort to a parametric framework. As we will see, the relative dimension of *m *with respect to *n *is not an issue in a Bayesian nonparametric framework, and the expected number of new genes that will be discovered in *m *further reads is monotone increasing with respect to *m*.

The application of Bayesian methods in this area of research is, to the authors' knowledge, quite modest even if the Bayesian learning scheme is very well suited for making predictions with EST data. An early contribution, based on a model for sampling from a finite population, is provided by Hill [9] where posterior estimates of the coverage are obtained. However, computational problems do not allow, in this approach, a direct and effective evaluation of the expected number of new species in a future sample. Recently, Lijoi *et al*. [10] have proposed new Bayesian nonparametric estimators for the problems a)-c) mentioned above. The prior distribution they employ is induced by a family of exchangeable *Gibbs *random partitions. See Pitman [11] for an interesting review of recent advances and applications of the theory of Gibbs random partitions. Their application to a Bayesian inferential framework is very useful since they provide a general scheme which encompasses some of the most notable nonparametric priors such as the Dirichlet and the two parameter Poisson-Dirichlet process. In this paper, we apply the general formulas derived in [10] with the two parameter Poisson-Dirichlet process as prior distribution. It will be seen that the resulting expressions can be evaluated exactly and do not need for any supplementary simulation scheme. Moreover, such a Bayesian approach does not incur in any problem for large values of *m *since all possible behaviors of future EST data are incorporated in the probabilistic model.

## Results and Discussion

### EST Datasets

The datasets we analyze consist of ESTs samples obtained from cDNA libraries from two different organisms: the amitochondriate protist *Mastigamoeba balamuthi *(non-normalized and normalized libraries, where the normalized library was prepared from the non-normalized library) and *Naegleria gruberi *libraries, prepared from cells grown under different culture conditions, aerobic and anaerobic. These data sets have been previously analyzed in [6], where a full account of their preparation is detailed. It is worth mentioning that our approach assumes full-length cDNA clones and high quality sequence reads.

Therefore, possible errors associated with the clustering procedure are not considered. For the statistical identification and evaluation of types of clustering errors one may incur in EST sequencing, the reader is referred to Wang *et al*. [12].

Specifically, each EST survey consists of *n *reads with *k *unique genes and corresponding frequencies *n*_1_,...,*n*_*k*_, i.e. *n*_*i *_is the number of tags displaying the *i*-th gene in the initial sample of size *n*. Clearly, $\sum_{i=1}^{k}n_{i}=n$. The reads can equivalently be clustered according to their level of expression, that is

(1) $$\begin{array}{ccc} r_{l}\equiv \sum_{i=1}^{k}I(\{n_{i}=l\}), & \text{for} & l=1,2,...,s \end{array}$$

where *I*(*A*) is an indicator of *A*: *I*(*A*) = 1 if *A *is true and 0 otherwise. Note that *s *represents the maximum level of expression among unique genes in the sample and that the number of positive *r*_*l*_'s is typically smaller than *s*.

Table 1 summarizes the four EST samples using the compact notation set in (1). For example, the survey of the *naeglaria *aerobic library produces *n *= 959 reads with *k *= 473 unique genes, which are clustered into 17 levels of expression 1, 2,...,12, 16, 17, 18, 27, 55. For the first level we have *r*_1 _= 346, meaning that 346 genes appear just once, that is *n*_1 _= *n*_2 _= … = *n*_346 _= 1. For the second level *r*_2 _= 57 implies that 57 genes appear twice and, hence, *n*_347 _= *n*_348 _= … = *n*_403 _= 2 and so on up to *r*_55 _= 1, which means that 1 gene is represented 55 times yielding *n*_473 _= 55.

**Table 1:** EST surveys information clustered into levels of expression

Library	*l*	1	2	3	4	5	6	7	8	9	10	11
**Naeglaria Aerobic**		346	57	19	12	9	5	4	2	4	5	4
**Naeglaria Anaerobic**		491	72	30	9	13	5	3	1	2	0	1
**Mastigamoeba Non-normalized**		378	33	21	9	6	1	3	1	1	1	0
**Mastigamoeba Normalized**		200	21	14	4	3	3	1	0	1	0	0

**Library**	*l*	**12**	**13**	**14**	**15**	**16**	**17**	**18**	**27**	**55**	*k*	*n*

**Naeglaria Aerobic**		1	0	0	0	1	1	1	1	1	473	959
**Naeglaria Anaerobic**		0	1	3	0	0	0	0	0	0	631	969
**Mastigamoeba Non-normalized**		0	1	0	5	0	0	0	0	0	460	715
**Mastigamoeba Normalized**		0	0	1	0	0	0	0	0	0	248	363

Source: Susko and Roger [6]

### Coverage, estimation of the number of new genes and discovery rate

We applied the Bayesian nonparametric method, detailed in the following section, to these datsets and obtained the following results. Denote the unknown proportion of genes (in the whole library) belonging to the *i*-th class by *p*_*i*_. Then, the coverage of the initial sample of size *n *is given by

(2) $$C=\sum_{i:n_{i}>0}p_{i},$$

which is precisely the proportion of unique genes represented in the initial sample. Our estimates for the coverage are 0.47 and 0.45 for the non-normalized (*n *= 715) and normalized (*n *= 363) *Mastigamoeba*, respectively. This means that, by virtue of the 'normalization', an initial sample of about half the size produces almost the same coverage. Moreover, we get 0.64 and 0.49 for the aerobic (*n *= 959) and anaerobic (*n *= 969) *Naegleria*, respectively: clearly, the tissue cultured aerobically achieves a remarkably higher coverage with an initial sample of the same size, which may lead to conclude that the sequencing of the aerobic tissue is more effective. However, such a finding could also be the consequence of a higher redundancy in the aerobic library. Finally, it is worth noting that our results for the coverage match exactly the ones obtained in [6], where the frequentist estimator described in [3] was exploited.

**Figure 1:**
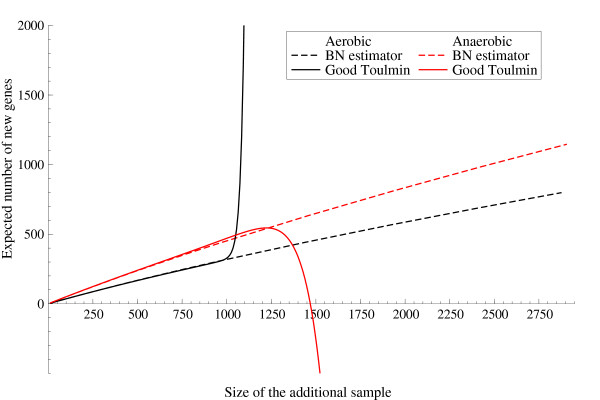
**Expected number of new genes: comparison with Good-Toulmin estimator**. Expected number of new genes in an additional sample for the *Naegleria gruberi *aerobic and anaerobic libraries arising from the application of the Good-Toulmin estimator and of the Bayesian nonparametric estimator.

Turning attention to predicting the outcomes of future sequencing for the libraries at issue, we focus on the expected number of new genes in an additional sample of size *m *and on the discovery rate. The first index provides an overall measure of redundancy with respect to the additional sample of size *m*, whereas the discovery rate predicts the trend at which the discovery probability decays as more and more reads come in. If one adopts a Bayesian nonparametric approach, these quantities can be estimated rigorously and exactly since such an approach is naturally designed for prediction. In contrast note that, as already anticipated, the Good-Toulmin estimator becomes highly variable and unstable if the size of the additional sample *m *is larger than the size of the initial sample *n*. In particular, the Good-Toulmin estimator often produces negative values as estimates for the number of new genes if *m *∈ (*n*, 2*n*) and almost always behaves badly for *m *> 2*n*. Such a phenomenon can be seen in Figure 1 for the two *Naegleria *libraries. In order to overcome these problems, frequentist methods typically give up the flexibility of the nonparametric approach and resort to parametric models, whose fit can be a delicate issue. For instance, Susko and Roger [6] resort to an approximated version of the Good-Toulmin estimator which assumes a parametric model for the expression levels *r*_*l*_.

In order to give a complete picture, it is important to accompany our point estimates with the 95% highest posterior density intervals, which represent the Bayesian counterpart to frequentist confidence intervals (see [13]). In Tables 2 and 3 the results arising by the application of the Bayesian nonparametric method are displayed.

**Table 2:** Estimates for the Mastigamoeba libraries

%*n*	*m*	Expected number of new genes in a additional sample of size *m*	Probability of discovering a new gene at the (*n *+ *m *+ 1)-th read
*Mastigamoeba *non-normalized			

50	358	180 ∈ (158 , 204)	0.481 ∈ (0.466 , 0.498)
100	715	346 ∈ (312 , 382)	0.452 ∈ (0.434 , 0.470)
150	1072	503 ∈ (458 , 550)	0.430 ∈ (0.411 , 0.449)
200	1430	654 ∈ (599 , 711)	0.412 ∈ (0.393 , 0.433)
250	1788	799 ∈ (734 , 866)	0.398 ∈ (0.379 , 0.419)
300	2145	939 ∈ (865 , 1015)	0.386 ∈ (0.367 , 0.407)

*Mastigamoeba *normalized			

50	182	94 ∈ (79 , 111)	0.493 ∈ (0.475 , 0.512)
100	363	180 ∈ (156 , 206)	0.456 ∈ (0.434 , 0.479)
150	544	260 ∈ (229 , 293)	0.428 ∈ (0.406 , 0.452)
200	726	336 ∈ (299 , 375)	0.406 ∈ (0.384 , 0.430)
250	908	408 ∈ (365 , 453)	0.389 ∈ (0.366 , 0.412)
300	1089	477 ∈ (428 , 528)	0.374 ∈ (0.351 , 0.398)

Non-normalized and normalized *Mastigamoeba *libraries: the first column provides the size of the additional sample in % of the size of the initial sample, the second the actual size of the additional survey, the third presents the expected number of new genes and the fourth the discovery probability. The estimates in the third and fourth column are accompanied by the 95% highest posterior density intervals.

**Table 3:** Estimates for the Naeglaria libraries

%*n*	*m*	Expected number of new genes in an additional sample of size *m*	Probability of discovering a new gene at the (*n *+ *m *+ 1)-th read
*Naegleria *aerobic			

50	480	162 ∈ (138 , 188)	0.318 ∈ (0.307 , 0.329)
100	959	307 ∈ (271 , 345)	0.290 ∈ (0.277 , 0.303)
150	1438	441 ∈ (394 , 488)	0.270 ∈ (0.257 , 0.282)
200	1918	566 ∈ (510 , 624)	0.254 ∈ (0.241 , 0.267)
250	2398	685 ∈ (619 , 751)	0.242 ∈ (0.229 , 0.255)
300	2877	798 ∈ (725 , 873)	0.231 ∈ (0.219 , 0.244)

*Naegleria *anaerobic			

50	484	231 ∈ (206 , 258)	0.450 ∈ (0.440 , 0.461)
100	969	440 ∈ (402 , 478)	0.412 ∈ (0.400 , 0.424)
150	1454	632 ∈ (583 , 683)	0.384 ∈ (0.371 , 0.397)
200	1938	812 ∈ (753 , 873)	0.362 ∈ (0.349 , 0.375)
250	2422	983 ∈ (915 , 1053)	0.344 ∈ (0.332 , 0.357)
300	2907	1146 ∈ (1069 , 1225)	0.330 ∈ (0.317 , 0.342)

*Naeglaria *aerobic and anaerobic libraries: the first column provides the size of the additional sample in % of the size of the initial sample, the second the actual size of the additional survey, the third presents the expected number of new genes and the fourth the discovery probability. The estimates in the third and fourth column are accompanied by the 95% highest posterior density intervals.

**Figure 2:**
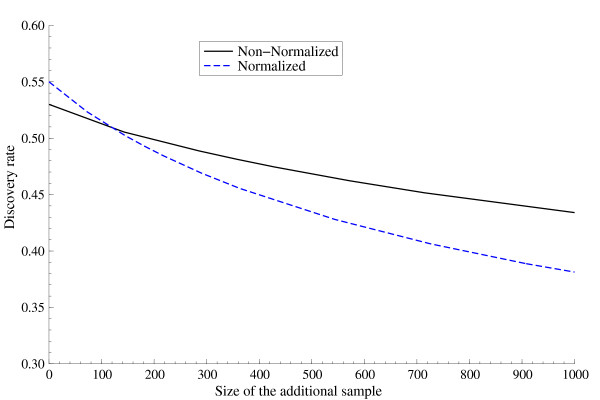
**Discovery rate**. Bayesian nonparametric estimates of the discovery rate associated to the non-normalized and normalized *Mastigamoeba *libraries.

As for the *Mastigamoeba *libraries, an interesting phenomenon takes place: the survey of the normalized library has achieved almost the same coverage (0.45) as the non-normalized library (0.47), but when considering an additional sample it exhibits a significantly faster decay in the discovery rate. Figure 2 compares the discovery rate for the two libraries. It is worth pointing out that our estimates predict that the discovery rates associated to both libraries coincide for *m *= 125 yielding a discovery probability of 0.508. For larger *m *the non-normalized exhibits a higher discovery rate. This implies that at some point also the estimates for the expected number of new genes in the additional sample will coincide: indeed, this is estimated to happen for *m *= 270, for which 137 new genes are predicted to be identified from both libraries. Hence, for *m *> 270 the expected number of new genes is systematically higher for the non-normalized library. For instance, if *m *= 1089, just 477 new genes are expected for the normalized library and 510 for the non-normalized. Taking *m *larger, at some point even the highest posterior density intervals will not overlap anymore. Such a behavior hints toward the fact that, in deciding whether to perform a 'normalization' protocol, the sizes of the samples to be drawn from the libraries is a variable to be taken into account.

**Figure 3:**
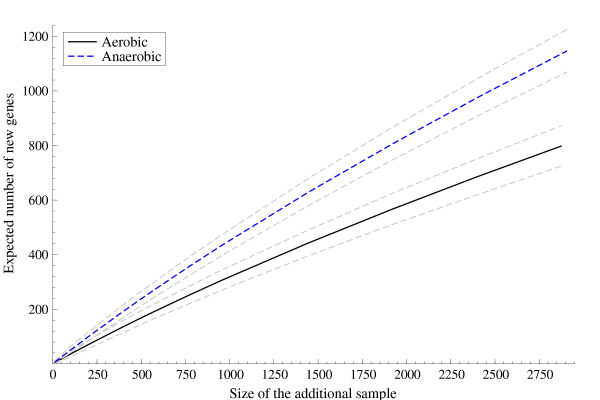
**Expected number of new genes**. Expected number of new genes in an additional sample and corresponding 95 % highest posterior density intervals for the *Naegleria gruberi *aerobic and anaerobic libraries arising from the application of the Bayesian nonparametric method.

As for the *Naegleria *libraries the behavior is apparent in the sense that the anaerobic library systematically produces more new genes and the discovery probability is sensibly higher at the considered levels of *m*. Note that the aerobic library presents a slightly slower decay rate but an extremely large *m *is required for matching the expected number of genes of the anaerobic one. Figure 3 displays the estimated decay rate of the discovery probability for both libraries with the corresponding 95% highest posterior density intervals.

### A Bayesian nonparametric methodology

The primary aim of the Bayesian approach to inference is prediction and Bayesian methods are tailored for conveying the available information into prediction. In particular, for EST sequencing, the main problem of frequentist methods is represented by the difficulty of incorporating not yet observed unique genes into the model. This can then produce unpleasant behaviors of estimators such as the one exhibited by the Good-Toulmin estimator discussed before. In contrast, the Bayesian nonparametric approach naturally incorporates the fact that further sequencing will feature new unique genes and leads to consistent predictions.

In our framework we are going to consider a sample of *n *EST data yielding *K*_*n *_distinct gene species with corresponding frequencies ***N ***= (*N*_1_,...,$N_{K_{n}}$). Clearly ***K***_*n *_∈ {1,...,*n*} and $\sum_{j=1}^{K_{n}}N_{j}=n$. Our basic model is the so-called Pitman's sampling formula [14] which consists of a probability distribution for *K*_*n *_and the frequencies ***N ***of the form

(3) $$Pr[K_{n}=k,N=n]=\frac{\prod_{i=1}^{k-1}\left(\theta +i\sigma \right)}{{\left(\theta +1\right)}_{n-1}}\prod_{j=1}^{k}(1-\sigma )n_{j}-1$$

where *σ *∈ (0, 1), *θ *> 0, ***n ***= (*n*_1_,...,*n*_*k*_) and (*a*)_*n *_= *a*(*a *+ 1) … (*a *+ *n *- 1) is the ascending factorial with (*a*)_0 _≡ 1. Formula (3) is a generalization of the famous Ewens' sampling formula [15] which can be recovered by letting *σ *tend to zero and it represents a important formula in modern probability theory. See [11]. Recently, it has found many interesting applications for bacterial taxonomy [16], clustering of microarray gene expression data [17], mixture models [18], linguistics [19], among others.

In a Bayesian nonparametric setting, one alternatively obtains model (3) by selecting the two parameter Poisson-Dirichlet process as a prior for the genes proportions within the library. This clearly makes the sequence of tags *exchangeable*, thus implying that the order of appearance of the tags does not influence probability assessments. Such an assumption, which constitutes the Bayesian analog of the frequentist assumption of independent and identically distributed data, is clearly reasonable in the context of EST sequences. Note that we implicitly assume that the sequence of tags can be extended to infinity. However, the size of the library represents an upper bound for the number of unique genes that will be observed and it is always finite: hence, all the estimates we are going to obtain will be finite.

As mentioned before, the Bayesian nonparametric approach has the advantage of yielding, in a straightforward way, predictive distributions for future observations given the data. Considering Pitman's sampling formula, the probability of detecting a new gene from a future observation, given a sample of *n *tags containing *k *distinct genes, is

(4) (*θ *+ *kσ*)/(*θ *+ *n*)

whereas the probability of re-observing the *j*-th unique gene coincides with

(5) (*n*_*j *_- *σ*)/(*θ *+ *n*)   *j *= 1,...,*k*.

See [11]. Hence, the coverage coincides with

(6) 1 - (*θ *+ *kσ*)/(*θ *+ *n*).

As already pointed out, in the analysis of ESTs one is also interested in evaluating: (i) the expected number of new genes that will be recorded in a further sample of size *m *and (ii) the discovery probability, which is the probability of observing a new gene in the (*n *+ *m *+ 1)-th draw, given the initial sample of size *n*. The basis for deriving estimators for these quantities is represented by the distribution of the number of new genes to be observed in an additional sample given the initial sample. Such a posterior probability, which can be seen as the predictive distribution for the outcome of additional *m *reads, is given by

(7) $$\begin{array}{c} P_{m}^{\left(k,n\right)}(j)=\frac{{\left(\theta +1\right)}_{n-1}}{{\left(\theta +1\right)}_{m+n-1}}\frac{\prod_{i=k}^{k+j-1}\left(\theta +i\sigma \right)}{\sigma j}\\ \times \frac{1}{j!}\sum_{i=0}^{j}{\left(-1\right)}^{i}\left(\begin{array}{c} j\\ i \end{array}\right){\left(n-(i+k)\sigma \right)}_{m} \end{array}$$

See [10] for details on its derivation. From (7) Bayes estimators (under quadratic loss function) for both the expected number of new genes and the discovery probability have been obtained, within general Gibbs random partition models, in [10]. The expected number of new genes observed in a future sample of size *m *coincides with

(8) $$E_{m}^{\left(k,n\right)}=\sum_{j=1}^{m}j\frac{{\left(k+\theta /\sigma \right)}_{j}}{{\left(\theta +n\right)}_{m}}\frac{1}{j!}\sum_{i=0}^{j}{\left(-1\right)}^{i}\left(\begin{array}{c} j\\ i \end{array}\right){\left(n-(i+k)\sigma \right)}_{m}$$

and the discovery probability turns out to be equal to

(9) $${\hat{D}}_{m}^{\left(k,n\right)}=\frac{\theta +\left[k+E_{m}^{\left(k,n\right)}\right]\sigma }{\theta +n+m}.$$

Moreover, the highest posterior density intervals can be derived in quite a straightforward way from (7). The only point left to discuss concerns the specification of the parameters (*σ*, *θ*). In order to avoid subjective inputs in the model, (*σ*, *θ*) is fixed according to an empirical Bayes rule which consists in choosing *σ *and *θ *that maximize (3) corresponding to the observed sample (*k*, *n*_1_,...,*n*_*k*_), i.e.

(10) $$(\hat{\sigma },\hat{\theta })=\arg\underset{\left(\sigma ,\theta \right)}{\max}\frac{\prod_{i=1}^{k-1}\left(\theta +i\sigma \right)}{{\left(\theta +1\right)}_{n-1}}\prod_{j=1}^{k}{\left(1-\sigma \right)}_{n_{j}-1}.$$

**Figure 4:**
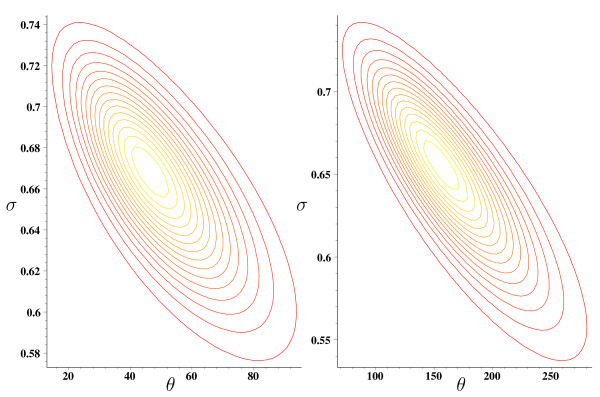
**Contour plots of Pitman's sampling formula**. Contour plots of Pitman's sampling formula, as a function of (*σ*, *θ*), corresponding to the two *Naegleria gruberi *datasets: aerobic (right) and anaerobic (left).

Figure 4 provides the contour plots corresponding to the two *Naegleria gruberi *datasets: the parameters maximizing (3) turned out to be ($\hat{\sigma },\hat{\theta }$) = (0.67, 46.3) for the aerobic case and ($\hat{\sigma },\hat{\theta }$) = (0.66, 155.5) for the anaerobic case. On the other hand, for the two *Mastigamoeba balamuthi *datasets (normalized and non-normalized) (10) yields ($\hat{\sigma },\hat{\theta }$) = (0.7, 57) and ($\hat{\sigma },\hat{\theta }$) = (0.77, 46), respectively. These parameters have been used for computing the estimators (8) and (9) for the 4 datasets, whose results are reported in the results and discussion section.

It is worth pointing out how the structure of the data influences the choice of the parameters (*σ*, *θ*). Indeed, the value of *θ *is linked to the number of distinct genes observed in the *n*-sample: the larger *k/n *the larger $\hat{\theta }$. On the other hand, the value of *σ *is determined by the configuration of the frequencies *n*_1_,...,*n*_*k*_. Moreover, one may note that, for a given value of *θ*, the expected number of new genes in (8) is an increasing function of *σ *: as *σ *increases one expects that a larger number of new genes is going to be observed in a further *m*-sample. This is also confirmed by the behavior of $E_{m}^{\left(k,n\right)}$ as *σ *varies. Figure 3 suggests an almost linear increase of $E_{m}^{\left(k,n\right)}$, as a function of *m*, and accordance with linearity is higher the closer *σ *is to 1. In contrast, when *σ *is low and close to 0 the function is concave and $E_{m}^{\left(k,n\right)}$ increases at a lower rate as *σ *increases.

In order to further evaluate the goodness of the estimation method, we implemented a cross-validation procedure. Given a dataset of size *n*, this consisted in randomly drawing without replacement a sub-sample of size *n*/2. On the basis of this sub-sample, we have fixed, according to (10), the values of (*σ*, *θ*). Then, we have computed the expected number of new genes in the remaining part of the original sample using (8) and checked whether the estimated number of new genes is close to the actually observed number of new genes. The outcome of such an analysis is satisfactory in the sense that, in the great majority of the performed experiments, the 95% highest posterior density intervals capture the actual number of new genes. Consider first the normalized *Mastigamoeba *library and a sub-sample of size *n *= 2 ≈ 182. For instance, when such a sub-sample consists of *k *= 144 distinct genes with expression levels *r*_1 _= 116, *r*_2 _= 24, *r*_3 _= 1, *r*_4 _= 2, *r*_7 _= 1, then the actual number of new genes in the remainder of the original sample is j* = 104. Basing on these data, the Bayesian nonparametric estimator $E_{181}^{\left(144,182\right)}$ yields an estimate of 101 with 95% highest posterior density interval (87, 117). As for the non-normalized *Mastigamoeba *library, sub-samples of size *n*/2 ≈ 358 were taken. For instance, a sub-sample consisting of *k *= 261 distinct genes with expression levels *r*_1 _= 213, *r*_2 _= 31, *r*_3 _= 4, *r*_4 _= 7, *r*_5 _= 1, *r*_7 _= 2, *r*_8 _= 3, implies that the remainder contains j* = 199 new genes, the number to be predicted. In such a case, the Bayesian nonparametric estimator $E_{357}^{\left(261,358\right)}$ predicts 210 new genes with 95% highest posterior density interval (187, 237).

## Conclusion

In this paper we have presented a Bayesian nonparametric approach, which relies on Pitman's sampling formula, for prediction problems arising in sequencing of EST libraries. This provides a fully probabilistic model which conveys, in a statistically rigorous way, the available information into prediction. No parametric assumption is made and the prior is fixed using an empirical Bayes approach. The resulting estimators are applied to four EST libraries and lead to interesting and coherent predictions of the outcome of additional sequencing. The arising information is of great value for researchers providing guidelines in: establishing the quality of a certain library; deciding whether to perform a normalization protocol; choosing whether to proceed with sequencing from a certain library; determining the size of an additional EST survey etc.

It is important to remark that our Bayesian nonparametric approach does not feature problems usually exhibited by frequentist methods. In particular, no *ad-hoc *adjustments or introduction of parametric components is necessary for predicting future reads if their number is larger than the initial survey. Finally, the estimators presented here can be easily adapted to take into account joint data from multiple libraries leading to Bayesian analogs of the estimators set forth in [6].

## Methods

Here we briefly describe how the estimators in (8) and (9) are derived by simplifying the expressions provided in [10]. In particular, one finds out that $E_{m}^{\left(k,n\right)}=\sum_{j=0}^{m}P_{m}^{\left(k,n\right)}(j)$, where the $P_{m}^{\left(k,n\right)}(j)$'s are displayed in (7) and can be deduced from (8) in [10]. The further simplification yielding the expression of $E_{m}^{\left(k,n\right)}$ in (8) is obtained by observing that (*θ *+ 1)_*n*-1_/(*θ *+ 1)_*n *+ *m*-1 _= 1/(*θ *+ *n*)_*m *_and

$$\frac{\prod_{i=k}^{k+j-1}\left(\theta +i\sigma \right)}{\sigma^{j}}={\left(k+\theta /\sigma \right)}_{j}$$

As far as the determination of (9), note that

$${\hat{D}}_{m}^{\left(k,n\right)}=\sum_{j=0}^{m}P_{1}^{\left(k+j,m+n\right)}(1)P_{m}^{\left(k,n\right)}(j)$$

where $P_{1}^{\left(k+j,m+n\right)}$ (1) is the probability of observing a new gene at the (*n *+ *m *+ 1)-th draw given the in the previous sample, of size *n *+ *m*, there have been detected *k *+ *j *distinct genes. Hence, by virtue of the prediction structure associated with the two parameter Poisson-Dirichlet process as outlined in Section 3, one has $P_{1}^{\left(k+j,m+n\right)}$ (1) = (*θ *+ (*k *+ *j*)*σ*)/(*θ *+ *n *+ *m*). From this one deduces

$$\begin{array}{l} {\hat{D}}_{m}^{\left(k,n\right)}=\sum_{j=0}^{m}\frac{\theta +(k+j)\sigma }{\theta +n+m}P_{m}^{\left(k,n\right)}(j)\\ =\frac{\theta +k\sigma }{\theta +n+m}\sum_{j=0}^{m}P_{m}^{\left(k,n\right)}(j)+\frac{\sigma }{\theta +n+m}\sum_{j=0}^{m}jP_{m}^{\left(k,n\right)}(j) \end{array}$$

and one obtains the expression in (9) since $\sum_{j=0}^{m}P_{m}^{\left(k,n\right)}(j)=1$.

## Competing interests

The author(s) declares that that there are no competing interests.

## Authors' contributions

The three authors participated in equal way in the development of the proposed methodology and the writing of the paper. All authors read and approved the final manuscript.
